# Italian intersociety consensus on management of long covid in children

**DOI:** 10.1186/s13052-022-01233-6

**Published:** 2022-03-09

**Authors:** Susanna Esposito, Nicola Principi, Chiara Azzari, Fabio Cardinale, Giuseppe Di Mauro, Luisa Galli, Guido Castelli Gattinara, Valentina Fainardi, Alfredo Guarino, Laura Lancella, Amelia Licari, Enrica Mancino, Gian Luigi Marseglia, Salvatore Leonardi, Raffaella Nenna, Stefania Zampogna, Stefano Zona, Annamaria Staiano, Fabio Midulla

**Affiliations:** 1 Pietro Barilla Children’s Hospital, Pediatric Clinic, Department of Medicine and Surgery, University Hospital, University of Parma, Via Gramsci 14, 43126 Parma, Italy; 2 Università degli Studi di Milano, Milan, Italy; 3 Department of Health Sciences, Section of Pediatrics, University of Florence, Florence, Italy; 4 Pediatric and Emergency Unit Giovanni XXIII Pediatric Hospital, University of Bari, Bari, Italy; 5 Family Paediatrician, Local Health Unit, Caserta, Italy; 6 Paediatric Infectious Disease Unit, Meyer Children’s University Hospital, Department of Health Sciences, University of Florence, Florence, Italy; 7 IRCCS Bambino Gesù Children Hospital, Rome, Italy; 8 Department of Translational Medical Sciences, Section of Pediatrics, University Federico II of Naples, Naples, Italy; 9 Paediatric and Infectious Disease Unit, Academic Department of Pediatrics, Bambino Gesù Children’s Hospital, IRCSS, Rome, Italy; 10 Maternal and Child Department, IRCCS Foundation Policlinico “S. Matteo” di Pavia, Pavia, Italy; 11 Department of Maternal, Infantile and Urological Sciences, Sapienza University of Rome, Rome, Italy; 12 Pediatric Respiratory Unit, Department of Clinical and Experimental Medicine, San Marco Hospital, University of Catania, Catania, Italy; 13 Department of Pediatrics, Pugliese-Ciaccio Hospital of Catanzaro, Catanzaro, Italy; 14 Primary Health Care Department, Local Health Agency of Modena, Modena, Italy

**Keywords:** COVID-19, Long COVID, Mental health, Pediatric infectious diseases, SARS-CoV-2

## Abstract

**Background:**

Two sequelae of pediatric COVID-19 have been identified, the multisystem inflammatory syndrome in children (MIS-C) and the long COVID. Long COVID is much less precisely defined and includes all the persistent or new clinical manifestations evidenced in subjects previously infected by SARS-CoV-2 beyond the period of the acute infection and that cannot be explained by an alternative diagnosis. In this Intersociety Consensus, present knowledge on pediatric long COVID as well as how to identify and manage children with long COVID are discussed.

**Main findings:**

Although the true prevalence of long COVID in pediatrics is not exactly determined, it seems appropriate to recommend evaluating the presence of symptoms suggestive of long COVID near the end of the acute phase of the disease, between 4 and 12 weeks from this. Long COVID in children and adolescents should be suspected in presence of persistent headache and fatigue, sleep disturbance, difficulty in concentrating, abdominal pain, myalgia or arthralgia. Persistent chest pain, stomach pain, diarrhea, heart palpitations, and skin lesions should be considered as possible symptoms of long COVID. It is recommended that the primary care pediatrician visits all subjects with a suspected or a proven diagnosis of SARS-CoV-2 infection after 4 weeks to check for the presence of symptoms of previously unknown disease. In any case, a further check-up by the primary care pediatrician should be scheduled 3 months after the diagnosis of SARS-CoV-2 infection to confirm normality or to address emerging problems. The subjects who present symptoms of any organic problem must undergo a thorough evaluation of the same, with a possible request for clinical, laboratory and / or radiological in-depth analysis in case of need. Children and adolescents with clear symptoms of mental stress will need to be followed up by existing local services for problems of this type.

**Conclusions:**

Pediatric long COVID is a relevant problem that involve a considerable proportion of children and adolescents. Prognosis of these cases is generally good as in most of them symptoms disappear spontaneously. The few children with significant medical problems should be early identified after the acute phase of the infection and adequately managed to assure complete resolution. A relevant psychological support for all the children during COVID-19 pandemic must be organized by health authorities and government that have to treat this as a public health issue.

**Supplementary Information:**

The online version contains supplementary material available at 10.1186/s13052-022-01233-6.

## Background

Children with acute severe acute respiratory syndrome coronavirus 2 (SARS-CoV-2) infection, even when symptomatic, generally clear the virus and recover within few days [1]. Risk of hospitalization and death is quite low. As of October 10, 2021, in the USA 6,396,278 child COVID-19 cases have been reported, 16.6% of all the available cases. Among them, only 0.1–2.0% were hospitalized and 0.00–0.03% died [2]. However, in some children the symptoms attributed to COVID-19 do not resolve quickly and can persist for several days or reappear after weeks or months. Two sequelae of pediatric COVID-19 have been identified, the multisystem inflammatory syndrome in children (MIS-C) and the long COVID, otherwise named post-acute sequelae of COVID-19, chronic COVID syndrome and post-COVID condition or syndrome [3, 4].

MIS-C is better characterized than long COVID. It is an acute and potentially fatal condition that occurs after 2 to 6 weeks after SARS-CoV-2 infection in less than 0.1% of pediatric COVID-19 cases and that is associated with marked immune activation and many potential mechanisms of immunopathogenesis [5]. Children with MIS-C present with several multisystemic manifestations, among which those involving the gastrointestinal tract, the skin and the mucous membranes, and the cardiovascular system are the most common. The latter are the most dangerous as vasogenic shock, myocardial dysfunction or infarction, coronary artery dilation or aneurysm, and arrhythmias can lead to admission to the intensive care unit and, although rarely, to death [6]. For some aspects, the clinical picture of MIS-C resembles that of Kawasaki disease (KD), especially in cases with shock, toxic shock syndrome, and macrophage activation syndrome [7]. As for KD, early identification of suspected cases is essential to assure a favorable prognosis. Prompt supportive care and drug therapy, mainly based on immunomodulatory drugs, are generally effective [8].

Long COVID is a different condition that is much less precisely defined than MIS-C and that has quite different clinical manifestations and therapeutic approach. It includes all the persistent or new clinical manifestations evidenced in subjects previously infected by SARS-CoV-2 beyond the period of the acute infection and that cannot be explained by an alternative diagnosis. Symptoms vary from those involving the mental health and the quality of life to those regarding the respiratory, cardiovascular, renal, and neurological systems. Long COVID has been largely studied in adults, mainly those hospitalized [9–23], but the lack of a clear definition of the syndrome, mainly because of absence of agreement on the type and duration of symptoms that justify the diagnosis, did not allow to precisely define long COVID characteristics. True prevalence, detailed clinical manifestations, long-term medical, social and economic impact, and the best therapeutic approach are only roughly defined. However, regardless of the criteria used to evaluate long COVID in adults, in this population long COVID is very common and can cause significant long-term problems. In a recent systematic review of studies that have enrolled 250,351 survivors of COVID-19, it was found that after 1 month, between 2 and 5 months, and after 6 or more months from COVID-19 diagnosis, 54, 55, and 54% of individuals had at least one abnormal manifestation with major social, health and economic impact [18]. The relevance ascribed to the clinical and socio-economic consequences of LC is illustrated by the evidence that, in UK, NICE, despite the lack of definitive conclusions on long COVID characteristics, has developed detailed guidelines aimed at facilitating the early identification of cases, the evaluation of the severity of symptoms and the implementation of the most appropriate therapeutic and rehabilitative modalities [23].

Contrarily to adults, long COVID has been poorly studied in children and the present knowledge of this condition in this population is poor [24]. The lower incidence and severity of COVID-19 in children compared to adults may explain why a very low number of studies concerning the long-term effects of SARS-CoV-2 infection in the first years of age has been conducted. Furthermore, the results of the few available studies are often conflicting, and this does not help to acquire convincing data on pediatric long COVID. Prevalence and duration are not defined and whether long COVID can have a lifelong health impact is not definitively established in pediatric age. However, despite further studies are needed to definitively characterize pediatric long COVID and define how to approach this condition in children, some data recently collected seem to indicate that pediatric long COVID could have specific clinical characteristics that suggest a peculiar diagnostic and therapeutic approach [25, 26]. In this Intersociety Consensus, present knowledge on pediatric long COVID as well as how to identify and manage children with long COVID are discussed.

## Methods

This paper has been drawn up by the Italian Society of Pediatrics (SIP) on initiative of the Pediatric Infectious Diseases Technical Committee and the Italian Society of Infantile Respiratory Diseases (SIMRI), in collaboration with the Italian Society of Pediatric Infectious Diseases (SITIP), the Italian Society of Pediatric Allergology and Immunology (SIAIP), the Italian Society of Pediatric Emergency and Urgency (SIMEUP), and the Italian Society of Preventive and Social Pediatrics (SIPPS). PubMed database was searched from inception to the 15th November 2021 for papers on long COVID-19 in children and adolescents. Papers describing the risk of long COVID, its course of disease and the outcome in patients otherwise healthy or with underlying chronic diseases were included. After the literature search, a manuscript was developed by the Chief of the SIP Pediatric Infectious Diseases Technical Committee (SE). It was revised by one of the experts (NP) and then submitted to all the other pediatricians selected by the Scientific Societies. A total of 4 recommendations were developed. Clarifications, adaptations, and refinements of the recommendations were made after a first round and participants were asked to approve the recommendations in a second round during the following 2 weeks.

## Definition

Regarding definition, the National Institute for Health and Care Excellence (NICE) of UK included in long COVID two conditions, the ongoing symptomatic COVID-19 and the post COVID-19 syndrome, the first one when symptoms were documented between 4 and 12 weeks after the start of COVID-19, and the second one when symptoms were still present more than 12 weeks later [27]. The US Centers for Disease Control and Prevention [28] consider long COVID in presence of clinical manifestations occurring 4 or more weeks after infection and, very recently, the World Health Organization [29] has established that post COVID-19 condition occurs in individuals with a history of probable or confirmed SARS-CoV-2 infection, usually 3 months from the onset of COVID-19 with symptoms that last for at least 2 months and cannot be explained by an alternative diagnosis.

### Recommendation 1

Long COVID is a clinical condition that includes all pathological manifestations following the acute phase of SARS-CoV-2 infection and which cannot be attributed to causes other than SARS-CoV-2. Although it is not currently possible to precisely define the type and time limits of these manifestations, long COVID can be considered after 3 months from the diagnosis of SARS-CoV-2 infection in the presence of symptoms that last for at least 2 months and which cannot be explained by another diagnosis.

## Epidemiology of long COVID in children

Already after few months from pandemic declaration, it was reported that, like adults, children with previous COVID-19, even if asymptomatic, could present symptoms of disease suggesting long COVID. The first report of pediatric long COVID came from Sweden describing five children with a median age of 12 years suffering from persistent symptoms 6 to 8 months after SARS-CoV-2 infection diagnosis. All of them had had a mild disease and none had been hospitalized because of COVID-19, nevertheless none of them had regularly returned to school [30]. Soon after, an Italian group, in a study enrolling children with laboratory-confirmed SARS-CoV-2 infection in the period from March to November 2020, showed that long COVID could be diagnosed in 66% (20 out of 30) and in 51.4% (35 out of 68) of patients assessed 60–120 days and 120 days or more after COVID-19 diagnosis, respectively [31]. Soon after, in UK, the Office for National Statistics calculated that, before February 2021, 12.9% of primary-school aged children and 15% of secondary-school aged children had had at least one symptom suggesting long COVID at 5 weeks post SARS-CoV-2 infection [32]. More recently, some studies confirmed these findings, although prevalence rates of long COVID reported by these studies varied significantly [33–46]. Low prevalence rates of long COVID were reported by Say et al. [36], Miller et al. [41], Radtke et al. [44] and Molteni et al. [45] who, examining children at 4 weeks from COVID-19 diagnosis, evidenced persisting symptoms in 8, 5, 9, and 4%, of the cases. Similar findings were reported by the UK Office for National Statistics that, after updating data previously published, in April 2021 revised estimates of pediatric long COVID prevalence, reducing them to 7.4% (primary-school aged) and 8.2% (secondary-school aged children) [47]. Moreover, in September 2021 these values were further revised downwards to 3.3% in primary-school aged children and 4.6% in those of secondary-school age for children at 4–8 weeks post-infection [48]*.*

However, significantly higher prevalence rates were reported by Smane et al. [34], Sterky et al. [37], Osmanov et al. [38], and Buonsenso et al. [39], and Stephenson et al. [40], who diagnosed long COVID in 30, 22, 24, 22, 87.1, and 66% of the children assessed at 12 weeks or more from COVID-19 onset. Several factors could justify these differences and explain why in a recent analysis of the available data, it was indicated that definitive conclusions on the prevalence and clinical characteristics of pediatric long COVID could not be drawn [29]. Studies were extremely heterogeneous due to significant differences in design, inclusion and exclusion criteria, duration of follow-up, and assessment of long-term clinical manifestations. Moreover, most of them had important methodological limitations. The total number of enrolled children was frequently too small to allow a reliable evaluation. Children without laboratory-confirmed COVID-19 were included [39, 43]. Collection of information was usually done with questionnaires and the responses of children or parents were not confirmed by clinical assessment or objective parameters [31, 37–45]. No data on pre-existing medical conditions, including mental health problems, were systematically reported. Cases with well-defined complications of COVID-19, such as pulmonary fibrosis or heart disease, and some MIS-C cases, especially the mildest, may have been erroneously included. Finally, only a few of the studies had a control group of children affected by similar infective disease to draw conclusions about specific long term sequelae of COVID-19 in children reliable and some studies reported potentially debatable data, as they were published without peer review [40–42].

Despite the limitations, a more in-depth evaluation of the available studies allows some observations that may be useful for a better knowledge of pediatric long COVID. All the studies, apart from the one by Denina et al. [46], have found that, although variable, a number of children has persistent or new clinical symptoms beyond the acute COVID-19 period. This finding definitively indicates that pediatric long COVID exists, and that it can be a clinical problem. Moreover, long COVID prevalence varied significantly according to the moment of symptoms assessment. Generally, it was low when assessment was performed at the end of the acute COVID-19 period or in the first weeks later [36, 41, 42, 45]. On the contrary, it was much higher when assessment was carried out at 12 or more weeks from infection [34, 37–39]. As in most of the pediatric COVID-19 cases infection and disease solve spontaneously in few days [1], the risk of persistent virus-related damage in children with COVID-19 is considered low and other mechanisms may play the major role in the pathogenesis of the clinical manifestations that emerge later. This supposition seems supported by the difference in long COVID prevalence between children and adults. Although prevalence of long COVID in children has been found lower than in adults regardless of the moment of symptom assessment [9–23], this phenomenon was much more evident when symptoms were assessed near the end of the acute COVID-19 period. Many symptomatic COVID-19 adult patients, particularly the elderly, have severe physical symptoms with long-term persistence revealing a relevant organ and body system damage which is difficult to repair and represents an essential component of the adult long COVID. This does not occur in the great majority of children and explains why children have low long COVID prevalence when evaluated after few weeks from infection.

Regarding risk factors for pediatric long COVID development, in some studies it has been shown that pediatric long COVID was more common in older children and adolescents [46], in females [40–42], in those with allergic problems [38] or with other chronic underlying disease [42], and in those with severe symptoms in the acute phase of COVID-19 [47]. A study specifically planned to evaluate risk factor for long COVID in children was recently carried out in Moscow, Russia [38]. A total of 518 children (mean age, 10.4 years; 52.1% girls) hospitalized for laboratory-confirmed COVID-19223–271 days before were enrolled. By means of a telephone interview with the parents/caregivers, it was shown that compared with children < 2 years of age, those aged 6–11 years and 12–18 years had a significant higher risk with odds ratio of 2.74 (95% confidence interval [CI], 1.37–5.75) and 2.68 (95% CI, 2.41–5.4), respectively. Furthermore, children with allergic diseases had an odd ratio of 1.67 (95% CI, 1.04–2.67) compared to children without. Finally, a trend toward a greater risk of long COVID was found in overweight/obese children. However, this study interviewed children who had COVID-19 several months before and this does not necessarily apply to children with recent infection.

### Recommendation 2

Although the true prevalence of long COVID in pediatrics is not exactly determined, it seems appropriate to recommend evaluating the presence of symptoms suggestive of long COVID near the end of the acute phase of the disease, between 4 and 12 weeks from this.

## Clinical manifestations and supposed pathogenesis of long COVID in pediatric age

Although with different frequency and duration between studies, the most common symptoms reported by children and adolescents with long COVID were headache (3 to 80%) and fatigue (3 to 87%), followed by sleep disturbances (2 to 63%), concentration difficulties (2 to 81%), abdominal pain (1 to 76%), myalgia or arthralgia (1 to 61%) [24]. In some studies, children have also referred persistent thoracic pain, stomachache, diarrhea, cardiac palpitations, skin irritation or lesions [24]. Particularly when more symptoms were coexisting and persistent, a severe limitation in daily functioning was reported causing great concern to pediatricians and parents [48–51]. The same factors previously listed to explain differences in long COVID prevalence may justify these variations.

Despite long COVID seems to be characterized by both physical and mental clinical manifestations, none of the symptoms considered alone or in combination appears to be specific of long COVID, as these manifestations can be referred by the general population even in absence of a defined disease. However, a chance to distinguish which symptoms can be attributed to the SARS-CoV-2 infection and which can have a different origin derives from the analysis of the studies that have compared children with SARS-CoV-2 infection with a group of children without infection matched for age, sex, and period of observation. Presently, data from 5 comparative studies are available [39–41, 44, 45]. Only in 3 of them [41, 42, 45], a higher prevalence of symptoms suggesting long COVID in children at 4 or 12 months was evidenced. Moreover, in 2 of them [41, 45] difference in symptoms’ prevalence between cases and controls at 4 weeks was not statistically significant and limited to 2–3%, suggesting that the risk of virus-related persisting symptoms in the first weeks after the end of the acute COVID-19 period is low in children, although in rare cases this may occur. Later evaluations (i.e., at about 12 weeks or later from infection) have confirmed that the risk of direct long-term consequences of SARS-CoV-2 infection are relatively uncommon. On the contrary, it was shown the majority of problems have a different origin and are probably related to the pandemic itself. Evidence in this regard can be found in the study by Stephenson et al. [42]. These authors studied pediatric long COVID administering detailed questionnaires to a group of 11–17-year old children, adolescents and young people after they have been tested for SARS-CoV-2 infection with reliable laboratory tests. A total of 3065 confirmed COVID-19 cases were compared to 3793 age-, sex- and geographically-matched SARS-CoV-2-negative controls. At 3 months after the test, symptoms regarding mental health, well-being, quality of life/functioning and fatigue were not only the most common symptoms, but they were also reported with the same frequency in all the children, regardless they were SARS-CoV-2-positive or -negative. Similar findings showing that several weeks after COVID-19 many children could suffer from mental symptoms were obtained by Blankenburg et al. [41]. These authors conducted a study enrolling a total of 1560 students (median age, 15 years), among whom 1365 (88%) were seronegative and 188 (12%) were seropositive. The children were asked to complete a validated questionnaire with 12 questions on the occurrence and frequency of relevant neurocognitive, pain and mood symptoms within the last 7 days before the survey. Each symptom was documented in about 35% of the students without any statistical difference between groups. Unhappiness (98.7%), tenseness (86.4%), listlessness (80.7%) and difficulties in concentrating (79.3%) were the most common.

Although a strict distinction between physical and mental health symptoms is debatable as mental stress can be associated with physical symptoms and long-term physical symptoms can cause mental health disorders, these findings indicate that, with time, physical symptoms due to SARS-CoV-2, even if persisting after 4 weeks from infection, tend to regress spontaneously or under treatment in few months, whereas mental problems can persist for a longer time. Several factors indicate that mental health problems depend on the stress conditions children underwent during pandemic. The association between infectious epidemics and the development of mental health problems in children does not surprise. Several studies carried out during previous severe epidemics, mainly the Ebola epidemic during which several children were orphaned, have shown that, together with medical problems, children could have severe psychological repercussions, with the development of frustration, worry or sadness, and feeling of being alone and being excluded by family or community [52]. In COVID-19 pandemic, the impact on the mental health of children and adolescents was also greater. To the problems strictly related to the infection, such as fear of contraction of the disease and to be hospitalized, and the grief for the loss of close relatives or friends, a major role in the development of mental disturbances has been played by the measures put in place worldwide by health authorities to reduce viral circulation and contain the number of COVID-19 cases [53, 54]. General lockdown was decided and maintained for a long time with very strong restrictions. This modified children lifestyle due to school closure, absence of outdoor activities, physical distancing, quarantine, isolation. Mental health of children has been severely compromised on account of increased anxiety, changes in their diets, school dynamics and education, fear of not knowing how to deal with emerging problems [54–57].

Development of mental health disfunctions during COVID-19 was found more common in older children and adolescents, in females and in those with previously diagnosed psychological problems [53, 54]. This is quite in agreement with what has been reported for pediatric long COVID, further suggesting that most of the clinical manifestations characterizing long COVID depend on the pandemic and not directly on the infection.

### Recommendation 3

Long COVID in children and adolescents should be suspected in presence of persistent headache and fatigue, sleep disturbance, difficulty in concentrating, abdominal pain, myalgia or arthralgia. Persistent chest pain, stomach pain, diarrhea, heart palpitations, and skin lesions should be considered as possible symptoms of long COVID.

## Approach to children with suspected long COVID

Present knowledge of the characteristics of pediatric long COVID are largely incomplete and this makes it difficult to indicate which is the most rational approach to this condition in children. However, waiting further studies capable of improving the knowledge in this regard, results of the studies presently available already allow some suggestions for a rational approach. Data indicate that pediatric COVID is a real problem [25, 26]. This means that all pediatric subjects with suspected or documented COVID-9 and their parents, even if asymptomatic at the time of diagnosis, must be informed of the natural course of the disease, of the fact that it generally heals within a few days of diagnosis but that in some cases the symptoms may persist for more than 4 weeks or, if disappeared, reappear with the same or with different characteristics. This because experts’ experience has documented that absence from or poor performance in education may be associated with poor outcomes for children and young people with symptoms of long COVID [58]. Moreover, as adolescents, females, children with underlying disease, including mental health, are at increased risk, particular attention must be paid to these subjects.

The results of the studies indicate that in the period immediately following the acute phase of COVID-19, practically between 4 and 12 weeks from diagnosis, only a small number of children has symptoms and that most of them are physical symptoms. Mental symptoms are less common. This suggests that all the children with previous suspected or documented COVID-19 regardless of the clinical manifestations and severity of disease must be checked by the primary care pediatrician during this period. The subsequent approach to the individual case will have to be evaluated on the basis of the present symptoms. According to the suggestions of the NICE, a questionnaire containing questions on the most common symptoms of pediatric long COVID should be used to obtain detailed information on long COVID development [58]. Supplementary Table 1 describes a useful questionnaire for the evaluation of long COVID in pediatric age. Children without clinical manifestations suggesting long COVID should be discharged without any immediate investigation but with the recommendation of a new visit in case of development of symptoms of any type. Partnership between families and pediatricians are a priority for better care [59]. Children with physical symptoms must be evaluated according to the characteristics of the clinical manifestations. The depth and type of investigations to be requested will depend on the clinical relevance and type of manifestations. As physical symptoms can indicate the involvement of several organs and body systems, it seems likely that several different physicians can be involved. These cases, after the first evaluation by the primary care pediatrician, will have to be followed with customized programs according to the clinical situation. For the few children that at 4–12 weeks from COVID present mental problems, the role of parents remains essential. They must offer lots of love and affection, establish and maintain the usual daily routine as much as possible, and be positive reassuring on the positive solution of al the pandemic-related problems. When mental problems are very severe and when they persist beyond the 12 weeks, psychological intervention with continuous psychological support becomes mandatory [56].

### Recommendation 4

Primary care pediatricians should visit all subjects with a suspected or a proven diagnosis of SARS-CoV-2 infection after 4 weeks to check for the presence of symptoms of previously unknown disease. In any case, a further check-up by the primary care pediatrician should be scheduled 3 months after the diagnosis of SARS-CoV-2 infection to confirm normality or to address emerging problems. The subjects who present symptoms of any organic problem must undergo a thorough evaluation of the same. Children and adolescents with clear symptoms of mental stress will need to be followed up by existing local services for problems of this type.

## Conclusions

Pediatric long COVID is a clinical problem that involve a relevant proportion of children. In a minority of cases, it can be considered a consequence of the viral infection. Prognosis of these cases is generally good as in most of them symptoms disappear spontaneously. The few children with significant medical problems should be early identified after the acute phase of the infection and adequately managed to assure complete resolution. Most of persistent or emerging symptoms of long duration regard mental health and do not depend on the infection but are part of the tremendous stress due to the restrictions put in place by health authorities to reduce COVID-19 incidence. It seems clear that a relevant psychological support for all the children during COVID-19 pandemic must be organized by health authorities and government that have to treat this as a public health issue.

Table 1 summarizes recommendations from our Intersociety Consensus on identification and management of children and adolescents with long COVID. However, more knowledge on the characteristics of the pediatric long COVID is needed to assure a more effective approach to the individual patient and studies with appropriate control groups are mandatory. As all the available data have been collected during circulation of the wild SARS-CoV-2, the role of variants, including the presently most common, the delta variant and the omicron, must be evaluated. This will not, however, be possible until a standardized definition of pediatric long COVID is developed. In addition, with a standardized definition of long COVID it will be possible to evaluate the impact of COVID vaccines for children and adolescents in reducing the burden of long COVID in pediatric age.

**Table 1:** Recommendations on identification and management of children and adolescents with long COVID

***Recommendation 1.*** Long COVID is a clinical condition that includes all pathological manifestations following the acute phase of SARS-CoV-2 infection and which cannot be attributed to causes other than SARS-CoV-2. Although it is not currently possible to precisely define the type and time limits of these manifestations, long COVID can be considered after 3 months from the diagnosis of SARS-CoV-2 infection in the presence of symptoms that last for at least 2 months and which cannot be explained by another diagnosis.	
***Recommendation 2.*** Although the true prevalence of long COVID in pediatrics is not exactly determined, it seems appropriate to recommend evaluating the presence of symptoms suggestive of long COVID near the end of the acute phase of the disease, between 4 and 12 weeks from this.	
***Recommendation 3.*** Long COVID in children and adolescents should be suspected in presence of persistent headache and fatigue, sleep disturbance, difficulty in concentrating, abdominal pain, myalgia or arthralgia. Persistent chest pain, stomach pain, diarrhea, heart palpitations, and skin lesions should be considered as possible symptoms of long COVID.	
***Recommendation 4.*** Primary care pediatricians should visit all subjects with a suspected or a proven diagnosis of SARS-CoV-2 infection after 4 weeks to check for the presence of symptoms of previously unknown disease. In any case, a further check-up by the primary care pediatrician should be scheduled 3 months after the diagnosis of SARS-CoV-2 infection to confirm normality or to address emerging problems. The subjects who present symptoms of any organic problem must undergo a thorough evaluation of the same. Children and adolescents with clear symptoms of mental stress will need to be followed up by existing local services for problems of this type.	

## Supplementary Information

**Additional file 1: Supplementary Table 1.** Questionnaire for the evaluation of long COVID in pediatric age.

## Data Availability

All data generated during this study are included in the published article.

## Abbreviations

CI: Confidence interval
KD: Kawasaki disease
MIS-C: Multisystem inflammatory syndrome
SARS-CoV-2: Severe acute respiratory syndrome coronavirus
