# Supplementary material for: Italian intersociety consensus on management of long covid in children
Source: Ital J Pediatr. 2022 Mar 9;48:42. doi: 10.1186/s13052-022-01233-6 (PMC8905554; doi:10.1186/s13052-022-01233-6)
Supplement: Supplementary file 1 — Additional file 1: Supplementary Table 1. Questionnaire for the evaluation of long COVID in pediatric age. [file 13052_2022_1233_MOESM1_ESM.docx]

**PARTICIPANT IDENTIFICATION** #: [ ][ ][ ][ ][ ]-[ ][ ][ ][ ]

SURVEY TIMEPOINT: 3m [ ] 6m [ ] 12m [ ] 24m [ ] 36m [ ]

Survey completed: ❑ Self-assessment ❑ Staff/research led assessment Completed by ❑ Telephone ❑ Post ❑ Clinic ❑ Online

# The questions were answered by the:


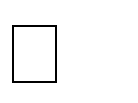
❑ Young person/child Parent or carer ❑ Other If *other*, please specify

# Your permission to proceed

Thank you for coming this far. Now please read the statements below, and initial the boxes if you are happy to go ahead.

| PLEASE MARK YOUR INITIALS AGAINST EACH STATEMENT WITH WHICH YOU AGREE: | *Add your Initials or tick the box:* | |
| --- | --- | --- |
| I give my consent for the information I provide. |  |  |
| I would like to continue to be sent this survey via email, post or to be contacted via telephone follow-up.  If yes, please enter your contact details here:  E-mail: Mobile phone number: Home telephone number: | YES | NO |
|  |  |  |
| Please enter your details  Your first name: Surname: Town/City of residence: Postcode: Your signature:  If you are completing the survey on behalf of your child please also enter your details below:  Your first name: Surname: Your signature: | | |

Local hospital/Primary care clinic ID:

| **1. About you (if the survey is completed by an adult or carer, all questions relates to the child)** |
| --- |
| **Sex/Gender:** ❑ Male ❑ Female ❑ Non-binary ❑ Prefer not to say  ***Ethnicity (tick all that apply):*** ❑ White ❑ Arab ❑ Black ❑ East Asian ❑ South Asian ❑ West Asian  ❑ Latin American ❑ Aboriginal/first nation ❑ Other (specify): ❑ Prefer not to say  **How many members regularly live in your household, including yourself:** [_Number_] |
| **2. About your Covid-19**  (if the survey is completed by an adult or carer, all questions relates to the child) |
| **Date you completed this survey (DD/MM/YYYY):** [_D_][_D_]/[_M_][_M_]/[_2_][_0_][_2_][_Y_]  **What is your date of birth (DD/MM/YYYY):** [_D_][_D_]/[_M_][_M_]/[_2_][_0_][_Y_][_Y_] |
| **2a. Do you believe that you have had Covid-19?** ❑ Yes ❑ No ❑ Not sure, if no skip to question 2b.  **If yes, approximately, what day did you first experience symptoms of Covid-19?**  [_D_][_D_]/[_M_][_M_]/[_2_][_0_][_Y_][_Y_] ❑ No symptoms  **How were you diagnosed with Covid-19?**  Laboratory confirmed (positive test) Physician/doctor confirmed Self-diagnosed  **Estimated date of your most recent positive SARS-CoV-2 /Covid-19 test:**  [_D_][_D_]/[_M_][_M_]/[_2_][_0_][_2_][_Y_] ❑ Not applicable |
| **2b. What symptoms did you experience at onset of Covid-19 (cases)?** |
| **What symptoms did you experience in the first 14 days of your illness?**  (tick all that you experienced when you first became unwell) 🞏Fever ≥ 38oC 🞏Runny nose 🞏 Headache  🞏Sore throat 🞏Muscle pain 🞏Abdominal pain 🞏Vomiting 🞏Diarrhoea  🞏 Cough 🞏Shortness of breath 🞏 Fatigue 🞏 Pain on breathing 🞏Chest pain 🞏Loss or disturbed smell  🞏Loss or disturbed taste 🞏Confusion 🞏Brain fog* 🞏Other symptoms:  🞏No symptoms  *****Brain fog (often described as a feeling ‘foggy’, confusion, short term memory problems, indecisive, not being able to think clearly) |

|  |  |
| --- | --- |
|  |  |
|  |  |
|  |  |
|  |  |
|  |  |

| **3. Hospitalisation** |
| --- |
| **Have you been admitted to hospital due to this illness (Covid-19)?**  ❑ Yes ❑ No If yes complete the below, if no skip to question 4.  **Roughly at what date were you first admitted to hospital?** [_D_][_D_]/[_M_][_M_]/[_2_][_0_][_2_][_Y_]  **Roughly at what date were you first discharged from hospital?** [_D_][_D_]/[_M_][_M_]/[_2_][_0_][_2_][_Y_]  Did you spend any time in an Intensive Care Unit (P/ICU)? ❑ Yes ❑ No ❑ Not sure Did your receive oxygen (e.g. via a mask, or nose cannula)? ❑ Yes ❑ No ❑ Not sure Have you been re-admitted to hospital after the first acute illness? ❑ Yes ❑ No  If yes, how many times : [_Number_] please specify main reason/reasons: |
| **4. Covid-19 vaccination** |
| **Have you been vaccinated against Covid-19?** ❑ Yes ❑ No if yes complete the below, if no skip to question 5a.  **If yes, how many times have you had the Covid-19 vaccine?** [_Number_]  **Which type of Covid-19 vaccine did you receive (if different types indicate all those you have received):**  ❑ AstraZeneca ❑ Pfizer-BioNTech ❑ Imperial ❑ Janssens ❑ Moderna’s ❑ Sinopharm ❑ Sputnik V  ❑ Other (name): ❑ Not sure  **Estimated date of the most recent Covid-19 vaccine dose received:** [_D_][_D_]/[_M_][_M_]/[_2_][_0_][_2_][_Y_] |
| **5a. About your emotional wellbeing, social relationships and activities today compared to before illness onset.** |
| To answer the following questions, please **mark an X** on the lines below that match your opinion on the question   1. **Compared to before your illness onset, how much are you now doing/experiencing the following**   ***Eating Sleeping***  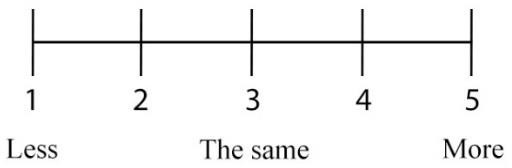 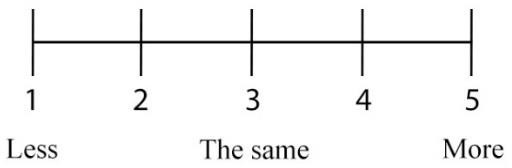  ❑ Unsure ❑ Unsure  ***Physical Activity Fatigue***  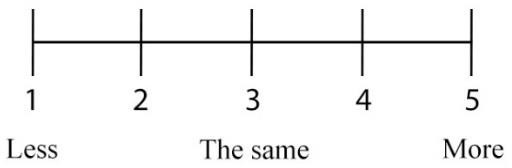 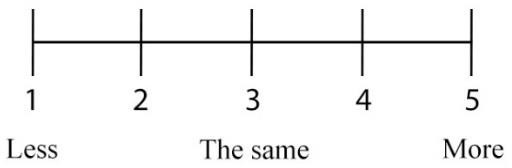  ❑ Unsure ❑ Unsure  ***Spending time with friends in-person Spending time with friends remotely (e.g., online, social media, texting)***  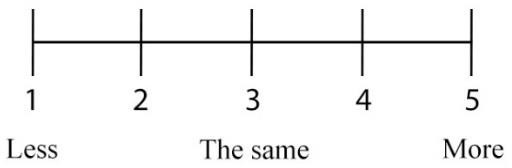 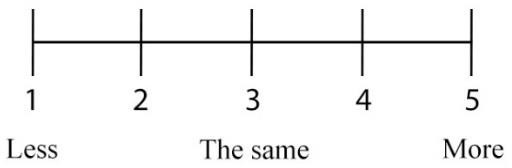  ❑ Unsure ❑ Unsure |

| ***Spending time watching TV, playing video/computer games, or using***  ***social media for educational purposes, including***  ***school/nursery work*** | ***Spending time watching TV, playing video/computer games, or using***  ***social media for non-educational purposes,*** |
| --- | --- |
| 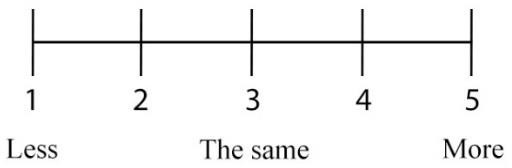  ❑ Unsure | 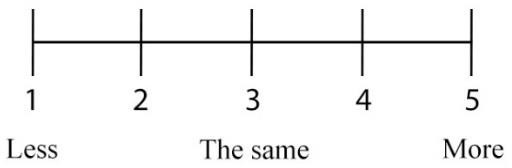  ❑ Unsure |
| ***Spending time outside*** | ***Attending nursery/school/university/work*** |
| 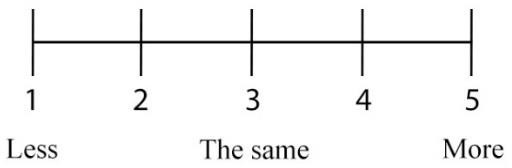  ❑ Unsure | 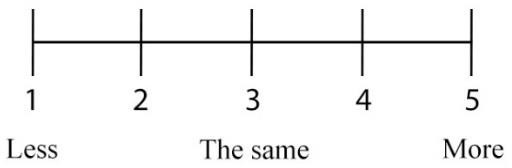  ❑ **Not applicable** |

| **… CONNECTEDNESS with others…** | **…EMOTIONS?** |
| --- | --- |
| 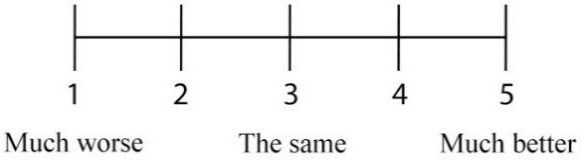  ❑ Unsure | 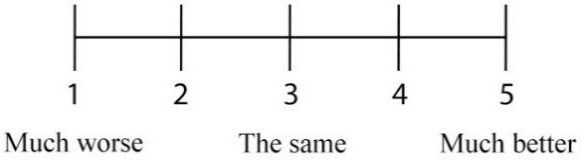  ❑ Unsure |
| **Compared to before your illness onset: Have there been changes in your …** | |
| **…BEHAVIOUR?** | **…RELATIONSHIPS, in how they get on with others?** |
| 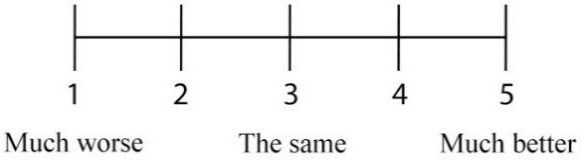  ❑ Unsure | 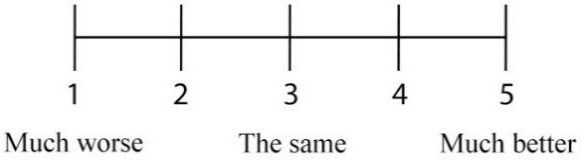  ❑ Unsure |

| **5b. About your state of health before you were diagnosed with Covid-19 (cases) OR other respiratory**  **illness (controls):** | | | | | |
| --- | --- | --- | --- | --- | --- |
| **Have you been physicians diagnosed or received treatment/support for any of the following prior to onset of your**  **illness that are still ongoing? (answer with a tick in the box)** | | | | | |
|  | **Yes** | **No** |  | **Yes** | **No** |
| Neurological/Neuro-disability |  |  | Immune system diseases |  |  |
| Gut/stomach problems |  |  | Genetic conditions |  |  |
| Heart diseases |  |  | Diabetes (if yes indicate type: □ Type 1 □ Type 2) |  |  |
| Respiratory diseases (not including asthma) |  |  | Other endocrine illness (not diabetes) |  |  |
| Asthma (doctors diagnosed) |  |  | Renal/Kidney problems |  |  |
| Allergic rhinitis/hay fever |  |  | Excessive weight or obesity |  |  |
| Food allergy |  |  | Malnutrition |  |  |
| Atopic dermatitis/Eczema |  |  | Depression |  |  |
| Rheumatological disease  *(e.g. arthritis, or inflammation of the joints)* |  |  | Anxiety |  |  |
| Sickle cell disease |  |  | HIV |  |  |
| Haematological disease *(other blood diseases)* |  |  | TB (tuberculosis) |  |  |
| Oncology (cancer, including lymphoma) |  |  | Other (please indicate): |  |  |
| **Were you born prematurely (<37 weeks)?** ❑ Yes ❑ No ❑ Not sure  **Have you ever sought support from a child /adolescent mental health/psychological services before the Covid-19 pandemic?** ❑ Yes ❑ No  **Prior to Covid-19 or other respiratory illness onset, how was your physical health in general?**  ❑ Very poor ❑ Poor ❑ Ok ❑ Good ❑ Very good  **Prior to Covid-19 or other respiratory illness onset, how would you describe your mental /psychological health in general?** ❑ Very poor ❑ Poor ❑ Ok ❑ Good ❑ Very good | | | | | |
| **6. About your current health** | | | | | |
| **Have you felt feverish recently?** ❑ Yes ❑ No ❑ Not sure  ***If yes indicate when you felt feverish (tick all that apply)***  ❑ Within the last 7 days ❑ 1-2 weeks ago ❑ >2-4 weeks ago ❑ >1-2 months ago ❑ >2-3 months ago  ❑ >3-6 months ago ❑ Since Covid-19/other respiratory illness onset  ***If yes, what was the most likely cause of your most recent feverish illness?***  ❑ Covid-19 ❑ Other respiratory infection (cough/cold/sore throat) ❑ TB  ❑ Stomach infection (diarrhea/vomiting) ❑ Urinary infection ❑ Other (specify):  ❑ Unknown ❑ Prefer not to say | | | | | |
| **How much do you agree with the following statement? “I have fully recovered from my infection”**  Please **mark an X** on the line below that match your opinion on the question as of **TODAY:**  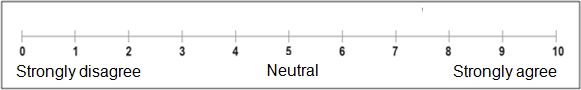 | | | | | |

| **7. We would like to know more about any problems you have had with feeling tired, weak or lacking in energy in the LAST MONTH**. Please answer ALL the questions by ticking the answer which applies to you most closely. If you have been feeling tired for a long while, then compare yourself to how you felt when you were last well | | | | | | |
| --- | --- | --- | --- | --- | --- | --- |
|  | | ***less than usual*** | | ***no more than usual*** | ***more than usual*** | ***much more than usual*** |
| Do you have problems with tiredness? | |  | |  |  |  |
| Do you need to rest more? | |  | |  |  |  |
| Do you feel sleepy or drowsy? | |  | |  |  |  |
| Do you have problems starting things? | |  | |  |  |  |
| Do you lack energy? | |  | |  |  |  |
| Do you have less strength in your muscles? | |  | |  |  |  |
| Do you feel weak? | |  | |  |  |  |
| Do you have difficulties concentrating? | |  | |  |  |  |
| Do you make slips of the tongue when speaking? | |  | |  |  |  |
| Do you find it more difficult to find the right word? | |  | |  |  |  |
|  | | ***better than usual*** | | ***no worse than usual*** | ***worse than usual*** | ***much worse than usual*** |
| How is your memory? | |  | |  |  |  |
| **8.** Within the **last seven days,** have you had any of these symptoms**,** which **were NOT present** before start of your Covid-19 / other respiratory illness (control group)? (Indicate if you have a symptom (tick Yes) or if you do not have a specific symptom (tick no)) | | | | | | |
| **Symptoms** | **Tick Yes/No** | | Indicate duration of symptoms (***in months***) and severity | | | |
| Nasal congestion / rhinorrhea | ❑ Yes ❑ No | | □ < 1 □ 1 □ 2 □ 3 □ 4 □ 5 □ 6 □ 7 □ 8 □ 9 □ 10 □ 11 □ ≥12  □ mild □ moderate □ severe □ very severe | | | |
| Difficulty breathing /chest tightness | ❑ Yes ❑ No | | □ < 1 □ 1 □ 2 □ 3 □ 4 □ 5 □ 6 □ 7 □ 8 □ 9 □ 10 □ 11 □ ≥12  □ mild □ moderate □ severe □ very severe | | | |
| Chest pain | ❑ Yes ❑ No | | □ < 1 □ 1 □ 2 □ 3 □ 4 □ 5 □ 6 □ 7 □ 8 □ 9 □ 10 □ 11 □ ≥12  □ mild □ moderate □ severe □ very severe | | | |
| Persistent cough | ❑ Yes ❑ No | | □ < 1 □ 1 □ 2 □ 3 □ 4 □ 5 □ 6 □ 7 □ 8 □ 9 □ 10 □ 11 □ ≥12  □ mild □ moderate □ severe □ very severe | | | |
| Problems with balance | ❑ Yes ❑ No | | □ < 1 □ 1 □ 2 □ 3 □ 4 □ 5 □ 6 □ 7 □ 8 □ 9 □ 10 □ 11 □ ≥12  □ mild □ moderate □ severe □ very severe | | | |
| Persistent muscle pain | ❑ Yes ❑ No | | □ < 1 □ 1 □ 2 □ 3 □ 4 □ 5 □ 6 □ 7 □ 8 □ 9 □ 10 □ 11 □ ≥12  □ mild □ moderate □ severe □ very severe | | | |
| Joint pain or swelling | ❑ Yes ❑ No | | □ < 1 □ 1 □ 2 □ 3 □ 4 □ 5 □ 6 □ 7 □ 8 □ 9 □ 10 □ 11 □ ≥12  □ mild □ moderate □ severe □ very severe | | | |
| Headache | ❑ Yes ❑ No | | □ < 1 □ 1 □ 2 □ 3 □ 4 □ 5 □ 6 □ 7 □ 8 □ 9 □ 10 □ 11 □ ≥12  □ mild □ moderate □ severe □ very severe | | | |
| Dizziness/ light headedness | ❑ Yes ❑ No | | □ < 1 □ 1 □ 2 □ 3 □ 4 □ 5 □ 6 □ 7 □ 8 □ 9 □ 10 □ 11 □ ≥12  □ mild □ moderate □ severe □ very severe | | | |

| Problems seeing/blurred vision | ❑ Yes ❑ No | | □ < 1 □ 1 □ 2 □ 3 □ 4 □ 5 □ 6 □ 7 □ 8 □ 9 □ 10 □ 11 □ ≥12  □ mild □ moderate □ severe □ very severe | | | |
| --- | --- | --- | --- | --- | --- | --- |
| Disturbed smell/Loss of smell | ❑ Yes ❑ No | | □ < 1 □ 1 □ 2 □ 3 □ 4 □ 5 □ 6 □ 7 □ 8 □ 9 □ 10 □ 11 □ ≥12  □ mild □ moderate □ severe □ very severe | | | |
| Disturbed taste/Loss of taste | ❑ Yes ❑ No | | □ < 1 □ 1 □ 2 □ 3 □ 4 □ 5 □ 6 □ 7 □ 8 □ 9 □ 10 □ 11 □ ≥12  □ mild □ moderate □ severe □ very severe | | | |
| Insomnia *(hard to fall asleep, hard to stay asleep)* | ❑ Yes ❑ No | | □ < 1 □ 1 □ 2 □ 3 □ 4 □ 5 □ 6 □ 7 □ 8 □ 9 □ 10 □ 11 □ ≥12  □ mild □ moderate □ severe □ very severe | | | |
| Hypersomnia *(excessive daytime sleepiness or prolonged nighttime*  *sleep)* | ❑ Yes ❑ No | | □ < 1 □ 1 □ 2 □ 3 □ 4 □ 5 □ 6 □ 7 □ 8 □ 9 □ 10 □ 11 □ ≥12  □ mild □ moderate □ severe □ very severe | | | |
| Tingling feeling/”pins and needles” | ❑ Yes ❑ No | | □ < 1 □ 1 □ 2 □ 3 □ 4 □ 5 □ 6 □ 7 □ 8 □ 9 □ 10 □ 11 □ ≥12  □ mild □ moderate □ severe □ very severe | | | |
| Fainting/black outs | ❑ Yes ❑ No | | □ < 1 □ 1 □ 2 □ 3 □ 4 □ 5 □ 6 □ 7 □ 8 □ 9 □ 10 □ 11 □ ≥12  □ mild □ moderate □ severe □ very severe | | | |
| Confusion/loss of concentration | ❑ Yes ❑ No | | □ < 1 □ 1 □ 2 □ 3 □ 4 □ 5 □ 6 □ 7 □ 8 □ 9 □ 10 □ 11 □ ≥12  □ mild □ moderate □ severe □ very severe | | | |
| Fatigue | ❑ Yes ❑ No | | □ < 1 □ 1 □ 2 □ 3 □ 4 □ 5 □ 6 □ 7 □ 8 □ 9 □ 10 □ 11 □ ≥12  □ mild □ moderate □ severe □ very severe | | | |
| Poor appetite | ❑ Yes ❑ No | | □ < 1 □ 1 □ 2 □ 3 □ 4 □ 5 □ 6 □ 7 □ 8 □ 9 □ 10 □ 11 □ ≥12  □ mild □ moderate □ severe □ very severe | | | |
| Diarrhea | ❑ Yes ❑ No | | □ < 1 □ 1 □ 2 □ 3 □ 4 □ 5 □ 6 □ 7 □ 8 □ 9 □ 10 □ 11 □ ≥12  □ mild □ moderate □ severe □ very severe | | | |
| Stomach/ abdominal pain | ❑ Yes ❑ No | | □ < 1 □ 1 □ 2 □ 3 □ 4 □ 5 □ 6 □ 7 □ 8 □ 9 □ 10 □ 11 □ ≥12  □ mild □ moderate □ severe □ very severe | | | |
| Feeling nauseous/persistent vomiting | ❑ Yes ❑ No | | □ < 1 □ 1 □ 2 □ 3 □ 4 □ 5 □ 6 □ 7 □ 8 □ 9 □ 10 □ 11 □ ≥12  □ mild □ moderate □ severe □ very severe | | | |
| Constipation | ❑ Yes ❑ No | | □ < 1 □ 1 □ 2 □ 3 □ 4 □ 5 □ 6 □ 7 □ 8 □ 9 □ 10 □ 11 □ ≥12  □ mild □ moderate □ severe □ very severe | | | |
| Palpitations (heart racing) | ❑ Yes ❑ No | | □ < 1 □ 1 □ 2 □ 3 □ 4 □ 5 □ 6 □ 7 □ 8 □ 9 □ 10 □ 11 □ ≥12  □ mild □ moderate □ severe □ very severe | | | |
| Variations in heart rate (tachycardia or bradycardia) | ❑ Yes ❑ No | | □ < 1 □ 1 □ 2 □ 3 □ 4 □ 5 □ 6 □ 7 □ 8 □ 9 □ 10 □ 11 □ ≥12  □ mild □ moderate □ severe □ very severe | | | |
| Skin rash | ❑ Yes ❑ No | | □ < 1 □ 1 □ 2 □ 3 □ 4 □ 5 □ 6 □ 7 □ 8 □ 9 □ 10 □ 11 □ ≥12  □ mild □ moderate □ severe □ very severe | | | |
| Other New Symptoms, if yes, specify: | | | indicate duration of symptoms (***in months***) and severity | | | |
|  | | | □ < 1 □ 1 □ 2 □ 3 □ 4 □ 5 □ 6 □ 7 □ 8 □ 9 □ 10 □ 11 □≥12  □ mild □ moderate □ severe □ very severe | | | |
|  | | | □ < 1 □ 1 □ 2 □ 3 □ 4 □ 5 □ 6 □ 7 □ 8 □ 9 □ 10 □ 11 □ ≥12  □ mild □ moderate □ severe □ very severe | | | |
|  | | | | | | |
| **9. Since having Covid-19, have you been diagnosed with any of the following?** | | | | | | |
|  | | **YES** | **NO** |  | **YES** | **NO** |
| Asthma | |  |  | Depression |  |  |
| Pulmonary embolism /micro emboli  *(PE, “Clot in lung”)* | |  |  | Anxiety |  |  |
|  |  |  |  | Diabetes If yes, ❑ Type 1 ❑ Type 2 |  |  |
| Kawasaki disease | |  |  | Shock / Toxic shock syndrome |  |  |
| Multisystem inflammatory syndrome  (MIS-C/PIMS-TS) | |  |  | Coagulopathy *(excessive bleeding or*  *clotting)* |  |  |
| Respiratory failure | |  |  | Kidney problems |  |  |
| Reduced lung function | |  |  | Other condition (if yes, specify): |  |  |
| Myocarditis *(inflammation of the heart muscle)* | |  |  |  |  |  |
|  | | | | | | |

|  | **10. Your overall health status** | |
| --- | --- | --- |
|  | TODAY BEFORE your   - We would like to know how good or bad your health is respiratory illness   - This line is numbered from 0 to 100   - 100 means the best health you can imagine. 0 means the worst health you can imagine.   - Please mark an X on the line that shows how   your health is TODAY and how it was BEFORE your illness.  Modified from © EuroQol Research Foundation. EQ-5D™ is a trade mark of the EuroQol Research Foundation | |
| **11. Health and wellbeing For Children and Teenagers from 8 to 17 years old If you/your child is under 8 years old skip to question 12**. | | |
| Describe your health **TODAY**  Under each heading, please tick the ONE box that describes your health TODAY | | |
| Mobility (walking about)  ❑ I have no problems walking about  ❑ I have some problems walking about  ❑ I have a lot of problems walking about | | Looking after myself  ❑ I have no problems washing or dressing myself  ❑ I have some problems washing or dressing myself  ❑ I have a lot of problems washing or dressing myself |


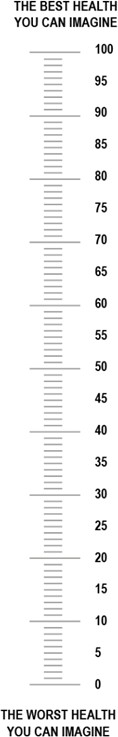

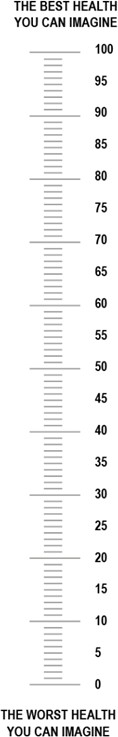


| Describe your health **TODAY**  Under each heading, please tick the ONE box that describes your health TODAY | |
| --- | --- |
| Doing usual activities (for example, going to school, hobbies, sports, playing, doing things with family or friends)  ❑I have no problems doing my usual activities  ❑ I have some problems doing my usual activities  ❑ I have a lot of problems doing my usual activities | Having pain or discomfort  ❑ I have no pain or discomfort  ❑ I have some pain or discomfort  ❑I have a lot of pain or discomfort |
| Feeling worried, sad or unhappy  ❑ I am not worried, sad or unhappy  ❑ I am a bit worried, sad or unhappy  ❑ I am very worried, sad or unhappy |  |
| Describe your health **BEFORE onset of your Covid-19**  Under each heading, please tick the ONE box that describes your health BEFORE onset | |
| Mobility (walking about)  ❑I had no problems walking about  ❑ I had some problems walking about  ❑I had a lot of problems walking about | Looking after myself  ❑I had no problems washing or dressing myself  ❑I had some problems washing or dressing myself  ❑ I had a lot of problems washing or dressing myself |
| Doing usual activities (for example, going to school, hobbies, sports, playing, doing things with family or friends)  ❑I had no problems doing my usual activities  ❑ I had some problems doing my usual activities  ❑ I had a lot of problems doing my usual activities | Having pain or discomfort  ❑I had no pain or discomfort  ❑ I had some pain or discomfort  ❑ I had a lot of pain or discomfort |
| Feeling worried, sad or unhappy  ❑I was not worried, sad or unhappy  ❑ I was a bit worried, sad or unhappy  ❑ I was very worried, sad or unhappy |  |
| **12. Please let us know of any additional comments about your Covid-19** | |
|  | |
| **End of survey** | |
| **Thank you for your time!** | |
